# Exon-Skipping Oligonucleotides Restore Functional Collagen VI by Correcting a Common *COL6A1* Mutation in Ullrich CMD

**DOI:** 10.1016/j.omtn.2020.05.029

**Published:** 2020-06-01

**Authors:** Sara Aguti, Véronique Bolduc, Pierpaolo Ala, Mark Turmaine, Carsten G. Bönnemann, Francesco Muntoni, Haiyan Zhou

**Affiliations:** 1 The Dubowitz Neuromuscular Centre, Molecular Neurosciences Section, Developmental Neurosciences Research and Teaching Department, Great Ormond Street Institute of Child Health, University College London, 30 Guilford Street, London WC1N 1EH, UK; 2 Neuromuscular and Neurogenetic Disorders of Childhood Section, National Institute of Neurological Disorders and Stroke, National Institutes of Health, Bethesda, Maryland MD 20892, USA; 3 Division of Biosciences, University College London, Gower Street, London WC1E 6BT, UK; 4 NIHR Great Ormond Street Hospital Biomedical Research Centre, London WC1N 1EH, UK; 5 Genetics and Genomic Medicine Research and Teaching Department, Great Ormond Street Institute of Child Health, University College London, London WC1N 1EH, UK

**Keywords:** Collagen VI, congenital muscular dystrophy, Ullrich muscular dystrophy, deep intronic mutation, exon skipping, antisense oligonucleotide, extra cellular matrix

## Abstract

Collagen VI-related congenital muscular dystrophies (COL6-CMDs) are the second most common form of congenital muscular dystrophy. Currently, there is no effective treatment available. COL6-CMDs are caused by recessive or dominant mutations in one of the three genes encoding for the α chains of collagen type VI (*COL6A1*, *COL6A2*, and *COL6A3*). One of the most common mutations in COL6-CMD patients is a *de novo* deep intronic c.930+189C > T mutation in *COL6A1* gene. This mutation creates a cryptic donor splice site and induces incorporation of a novel in-frame pseudo-exon in the mature transcripts. In this study, we systematically evaluated the splice switching approach using antisense oligonucleotides (ASOs) to correct this mutation. Fifteen ASOs were designed using the RNA-tiling approach to target the misspliced pseudo-exon and its flanking sequences. The efficiency of ASOs was evaluated at RNA, protein, and structural levels in skin fibroblasts established from four patients carrying the c.930+189C > T mutation. We identified two additional lead ASO candidates that efficiently induce pseudo-exon exclusion from the mature transcripts, thus allowing for the restoration of a functional collagen VI microfibrillar matrix. Our findings provide further evidence for ASO exon skipping as a therapeutic approach for COL6-CMD patients carrying this common intronic mutation.

## Graphical Abstract

**Figure:**
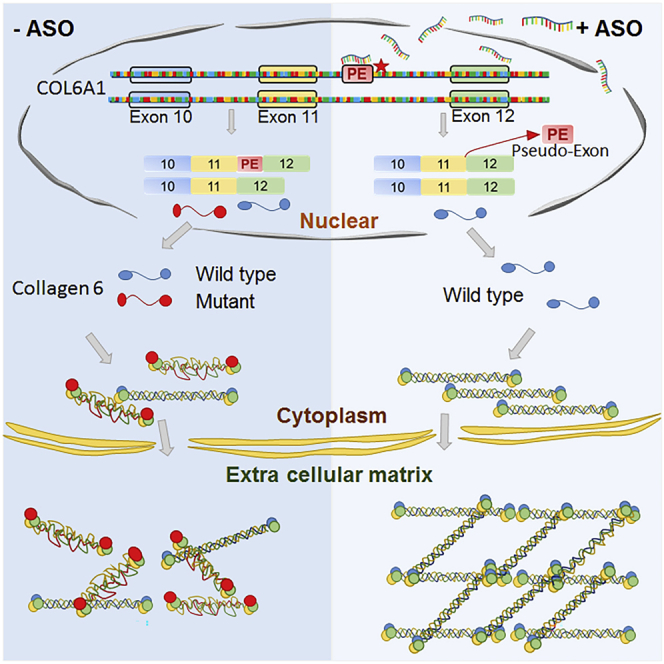

## Teaser

COL6-related congenital muscular dystrophies are caused by mutations in *COL6A* genes leading to the reduction of collagen VI protein in extracellular matrix. Zhou and colleagues report an exon-skipping antisense approach to successfully correct a common deep intronic mutation and restore functional collagen VI protein in cultured skin fibroblasts from patients. Keywords: collagen VI, congenital muscular dystrophy, Ullrich muscular dystrophy, deep intronic mutation, exon skipping, antisense oligonucleotide, extra cellular matrix

## Introduction

Congenital muscular dystrophy (CMD) is a term coined in 1908 that is used to identify a group of heterogeneous muscle wasting diseases with onset at birth or infancy.^1^ One of the main CMD subtypes is collagen VI-related congenital muscular dystrophies (COL6-CMDs). COL6-CMDs are caused by recessive or dominant mutations in one of the three genes encoding for the α chains of collagen type VI protein (collagen VI).^2^, ^3^, ^4^ α1 and α2 chains are encoded by *COL6A1* (NM_001848.3) and *COL6A2* (NM_001849.4), respectively, located on chromosome 21q22.3. The α3 chain is encoded by *COL6A3* (NM_004369.4) located on chromosome 2q27.^5^ The three α chains share the N-terminal and C-terminal globular domains connected by a short triple-helical domain consisting of Gly-X-Y repeating sequences, where X is often proline and Y is often hydroxyproline or hydroxylysine.^6^ Together, the three α chains assemble to form the large higher-order collagen VI protein that is an important component of the extracellular matrix (ECM). Before being secreted into ECM, collagen VI undergoes numerous assembly steps.^7^^,^^8^ This multi-step assembly process starts in the endoplasmic reticulum, where α chains combine to form triple-helix monomers in equal stoichiometry. Monomers are then associated in an anti-parallel manner to form dimers that subsequently assemble into tetramers. Tetramers undergo post-translational modifications and ultimately are secreted into the extracellular compartment, where they align in an interlinking end-to-end association to form a network of beaded microfibrils.^8^, ^9^, ^10^, ^11^ Collagen VI microfibrils are ubiquitously distributed throughout connective tissues, anchoring components of the basal lamina to the surrounding ECM.^12^ This function is crucial for signal transduction and cell integrity, particularly in skeletal muscle that continuously undergoes contraction-induced mechanical stress.^9^ In skeletal muscles, collagen VI is synthesized by the interstitial muscle fibroblasts and represents one of the major components of the ECM.^13^

COL6-CMDs range from the severe Ullrich CMD (UCMD; MIM #254090), via phenotypes of intermediate severity, to the milder Bethlem myopathy (BM; MIM #158810). UCMD was originally described by Otto Ullrich in the 1930s.^14^ Onset of UCMD is congenital or in early childhood and is characterized by progressive muscle weakness and wasting, scoliosis, distal joint hypermobility associated with proximal joint contractures, and progressive respiratory failure. UCMD patients may never acquire the ability to walk independently or the ability is lost in the first decade of life.^15^, ^16^, ^17^ UCMD is caused by both recessively and dominantly acting *COL6A1*, *COL6A2*, or *COL6A3* mutations.^3^^,^^18^, ^19^, ^20^, ^21^ More than 50% of UCMD cases harbor *de novo* dominant mutations, which are typically splice-site mutations or in-frame genomic deletions in the triple-helical domains, or glycine missense mutations affecting the Gly-X-Y collagenous motifs in the N-terminal part of the triple-helical domains. Mutations in this location render the mutant chain unable to perform tetrameric assembly as the basis of their dominant-negative effect.^22^ In the remaining UCMD cases, the disease is caused by recessive mutations in *COL6A* genes. Recessive mutations mainly lead to a premature stop codon resulting in nonsense-mediated mRNA decay. Because all the collagen VI α chains are required for the formations of functional tetramers, patients with recessive mutations are typically unable to produce extracellular collagen VI.^3^^,^^18^^,^^23^ In patients with dominant variants, mutant tetramers either fail to be secreted into the ECM, causing a reduction of collagen VI protein in ECM and retention in cytoplasm, and/or mutant tetramers are secreted into the ECM, but they show a reduced ability to associate with other tetramers to form the functional beaded microfibril network.^19^^,^^21^ The absence or reduction of functional collagen VI in the ECM leads to loss of mechanical anchoring between the matrix and the basement membrane.^9^^,^^24^

Currently, there is no effective treatment for COL6-CMD.^25^ The use of antisense oligonucleotide (ASO) has recently been explored as a therapeutic approach for COL6-CMD caused by mutations acting in a dominant-negative fashion. We and others have explored allele-specific silencing using ASO or siRNA to selectively suppress the expression of the mutant transcripts.^26^, ^27^, ^28^, ^29^ This approach is based on the fact that haploinsufficiency is not associated with disease.^3^^,^^23^^,^^30^^,^^31^ We previously reported the therapeutic potential of gapmer ASOs to selectively silence the mutant allele for a dominant UCMD mutation. The allele carrying the genomic deletion was successfully suppressed at transcript levels, leading to the restoration of functional collagen VI protein in the ECM.^27^

**Figure 1:**
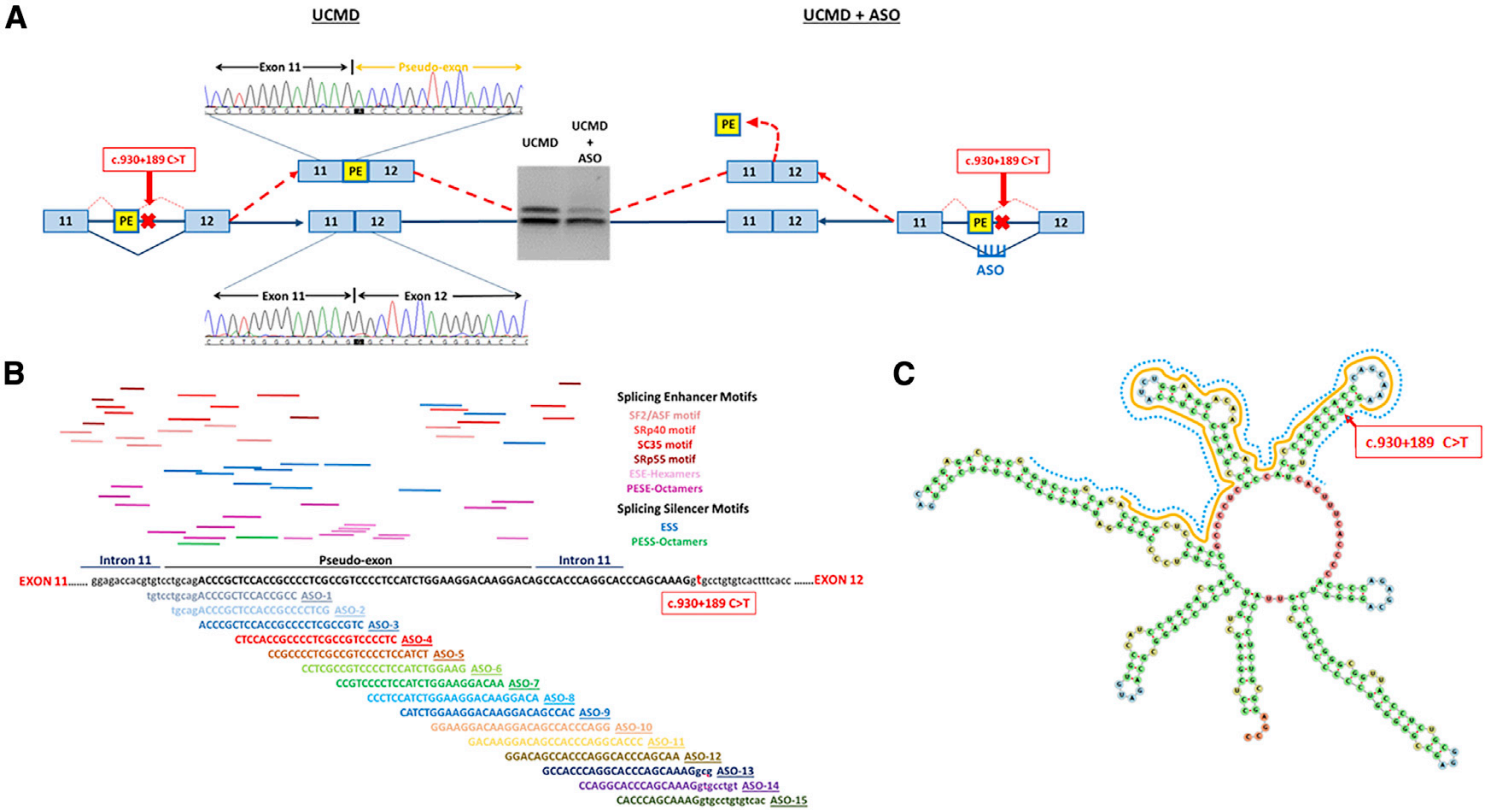
Exon-Skipping Strategy Using Antisense Oligonucleotide (A) The deep intronic mutation c.930+189C > T in *COL6A1* creates a cryptic splice donor site responsible for the insertion of an in-frame pseudo-exon (PE) between exons 11 and 12 in the mature transcripts, as confirmed by PCR and Sanger sequencing. The exon-skipping strategy designed to target the c.930+189C > T mutation to skip the PE is illustrated. (B) Schematic representation of PE and its flanking sequences. The top panel shows the potential splicing enhancer and splicing silencer motifs predicted by the Human Splicing Finder (http://www.umd.be/HSF/). The bottom panel illustrates the 15 ASOs designed to anneal to the PE and its flanking 5′ and 3′ splice sites. (C) M-fold of PE and its flanking 5′ and 3′ sites using RNAfold web server (http://rna.tbi.univie.ac.at//cgi-bin/RNAWebSuite/RNAfold.cgi). The PE is highlighted in yellow, and the region recognized by ASOs is indicated in blue.

In this study, we explore the ASO exon-skipping approach for a *de novo* recurrent deep intronic variant c.930+198C > T in *COL6A1* gene.^32^ This recently discovered variant is one of the most common dominant-negative mutations that cause UCMD. Patients carrying this mutation typically present with severe clinical features.^33^ *COL6A1* c.930+189C > T mutation creates a cryptic splice donor site in intron 11 that results in an in-frame insertion of a 72-bp pseudo-exon in the mature mRNA transcripts (Figure 1A).^33^ The localization of the pseudo-exon between wild-type exons 11 and 12 falls within the N-terminal triple-helical domain of *COL6A1*, where other assembly-competent dominantly acting mutations are also located. Thus, when the pseudo-exon including transcripts are translated, the mutant peptide containing α1 chain is capable of assembling with the other α2 and α3 chains to form mutant tetramers that are secreted in the extracellular space, where they subsequently interfere with collagen VI matrix assembly. Mutant tetramers fail to form the functional beaded microfibrils in the ECM, leading to reduction and mislocalization of the collagen VI protein. Phosphorodiamidate morpholino oligomer (PMO)-based exon-skipping ASOs, previously characterized by us, successfully suppressed the incorporation of the pseudo-exon in primary cultures of patient fibroblasts, although they did not achieve complete skipping even at high concentrations, indicating the need for further optimization.^32^

To enhance the skipping efficiency, systematically define the optimal sequence, and explore a different backbone chemistry, we designed a series of 2′-*O*-methyl (2′-OMe), and 2′-*O*-methoxyethyl (2′-MOE) ASOs using an RNA-tiling approach to target the entire aberrantly spliced intronic sequence and its flanking regions. This study was carried out in skin-derived fibroblast cultures from four patients carrying the c.930+189C > T mutation in *COL6A1*. Evaluation of ASOs on pseudo-exon skipping was performed at RNA and protein levels, and on microfibril structure in the ECM. Among the 15 ASOs screened in this study, we selected two of the most effective ASOs and further refined their design by testing different chemistry and lengths. We analyzed the efficiency of these ASOs in parallel with a previously published lead sequence synthesized in 2′-MOE chemistry (ASO-9).^32^ Two lead ASOs, a 20-mer ASO (ASO-5/6) and a 25-mer ASO (ASO-6), together with ASO-9, showed striking exon-skipping efficacy on correcting the mutant transcripts and restoring wild-type protein in ECM with near-normal ultrastructure of the collagen VI microfibril network.

## Results

### Design and Systematic Screening of ASOs to Skip the Pseudo-exon from the Mutant *COL6A1* Transcripts

**Figure 2:**
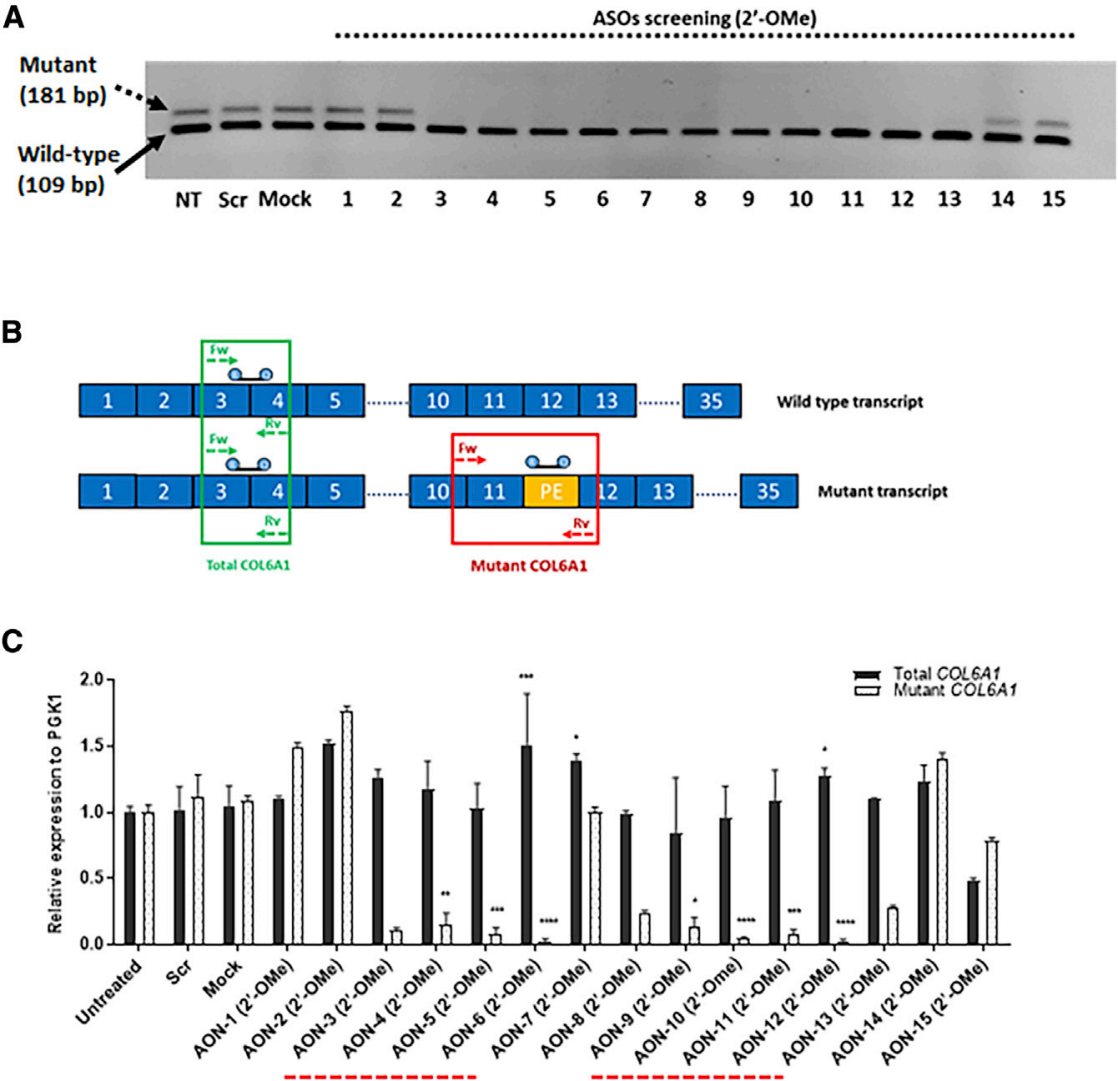
Evaluation of ASOs Specificity and Efficiency in Pseudo-Exon Skipping at the RNA Level (A) Gel electrophoresis of PCR products amplified in RNA samples isolated from UCMD fibroblasts treated with ASOs at 20 nM for 24 h with Lipofectamine transfection. (B) Schematic representation of quantitative real-time PCR assay. Two sets of primers with specific probes were used in the analysis. One set of primers with a specific probe complementary to the pseudo-exon (PE) to exclusively amplify the mutant transcripts. Another set of primers to amplify the total *COL6A1* transcripts, including both wild-type and mutant transcripts. (C) Quantitative real-time PCR was performed in RNA samples collected from UCMD fibroblasts treated with 20 nM ASOs after 24 h of transfection, using specific primers and probes. The two regions, where ASOs are capable of efficiently skipping the PE from the mutant transcripts, are underlined by a dashed line in red. Data were normalized to untreated samples and analyzed by one-way ANOVA and post-Bonferroni test. Data are presented as mean ± SD (∗p ≤ 0.05; ∗∗p ≤ 0.01; ∗∗∗p ≤ 0.001).

Fifteen ASOs were designed to anneal to the 72-bp pseudo-exon and its flanking 5′ and 3′ sequences, with each ASO 25-mer long at 5-nt intervals (Figures 1B and 1C). All ASOs were synthesized in 2′-OMe chemistry in the initial screening (Table 1). The evaluation of all 15 ASOs was carried out in skin-derived fibroblast cultures, established from four patients carrying the same mutation. ASOs were screened for efficiency at concentrations of 100 and 20 nM, respectively. The transcripts of wild-type and mutant alleles were amplified by RT-PCR and visualized in agarose gels. Striking skipping of the pseudo-exon was similarly observed at both concentrations, and the lower concentration, 20 nM, was hence selected for further experiments. Agarose gel analysis showed an evident effect on skipping the mutant transcripts after treatment with ASO-3 to ASO-13 at 20 nM (Figure 2A). To quantitatively assess the efficiency of different ASOs on pseudo-exon skipping, we performed quantitative real-time PCR on RNA samples collected from fibroblasts treated with 20 nM ASOs to specifically amplify the mutant and total (mutant + wild-type) *COL6A1* transcripts (Figure 2B). Our results showed two regions of the pseudo-exon sequence, targeted by ASO-3 to ASO-6 and ASO-9 to ASO-12, respectively, where ASOs successfully skipped the mutant transcripts with efficiencies over 90%, including 91.3% (±4.6) after ASO-5 treatment and 97.5% (±2.2) after ASO-6 treatment (Figure 2C).

**Table 1:** ASOs Sequences Used in This Study

Name	Sequence (5′–3′)	ASO Chemistry	ASO Length	GC%	Tm	Energy (Kcal/mol)
ASO-1	GGCGGUGGAGCGGGUCUGCAGGACA	2′-OMe	25	72	67.5	−4.1
ASO-2	CGAGGGGCGGUGGAGCGGGUCUGCA	2′-OMe	25	76	69.2	−3.5
ASO-3	GACGGCGAGGGGCGGUGGAGCGGGU	2′-OMe	25	80	70.8	−4.4
ASO-4	GAGGGGACGGCGAGGGGCGGUGGAG	2′-OMe	25	80	70.8	−4.4
ASO-5	AGAUGGAGGGGACGGCGAGGGGCGG	2′-OMe/2′-MOE	25	76	69.2	−1.7
ASO-6	CUUCCAGAUGGAGGGGACGGCGAGG	2′-OMe/2′-MOE	25	68	65.9	−5.6
ASO-7	UUGUCCUUCCAGAUGGAGGGGACGG	2′-OMe	25	60	62.6	−10.3
ASO-8	UGUCCUUGUCCUUCCAGAUGGAGGG	2′-OMe	25	56	61	−7.7
ASO-9	GUGGCUGUCCUUGUCCUUCCAGAUG	2′-OMe/2′-MOE	25	56	61	−0.9
ASO-10	CCUGGGUGGCUGUCCUUGUCCUUCC	2′-OMe	25	64	64.2	−2.7
ASO-11	GGGUGCCUGGGUGGCUGUCCUUGUC	2′-OMe	25	68	65.9	−6.9
ASO-12	UUGCUGGGUGCCUGGGUGGCUGUCC	2′-OMe	25	68	65.9	−5.7
ASO-13	CACCUUUGCUGGGUGCCUGGGUGGC	2′-OMe	25	68	65.9	−8.7
ASO-14	ACAGGCGCCUUUGCUGGGUGCCUGG	2′-OMe	25	68	65.9	−17
ASO-15	GUGACACAGGCGCCUUUGCUGGGUG	2′-OMe	25	64	64.2	−4.7
ASO-5/6	AGAUGGAGGGGACGGCGAGG	2′-MOE	20	70	60	0

Parameters listed in the table include ASO chemistry, length, GC content, melting temperature (Tm), and the minimum free energy expressed in Kcal/mol.

### Efficacy of ASO to Restore Collagen VI Protein Expression in the ECM

**Figure 3:**
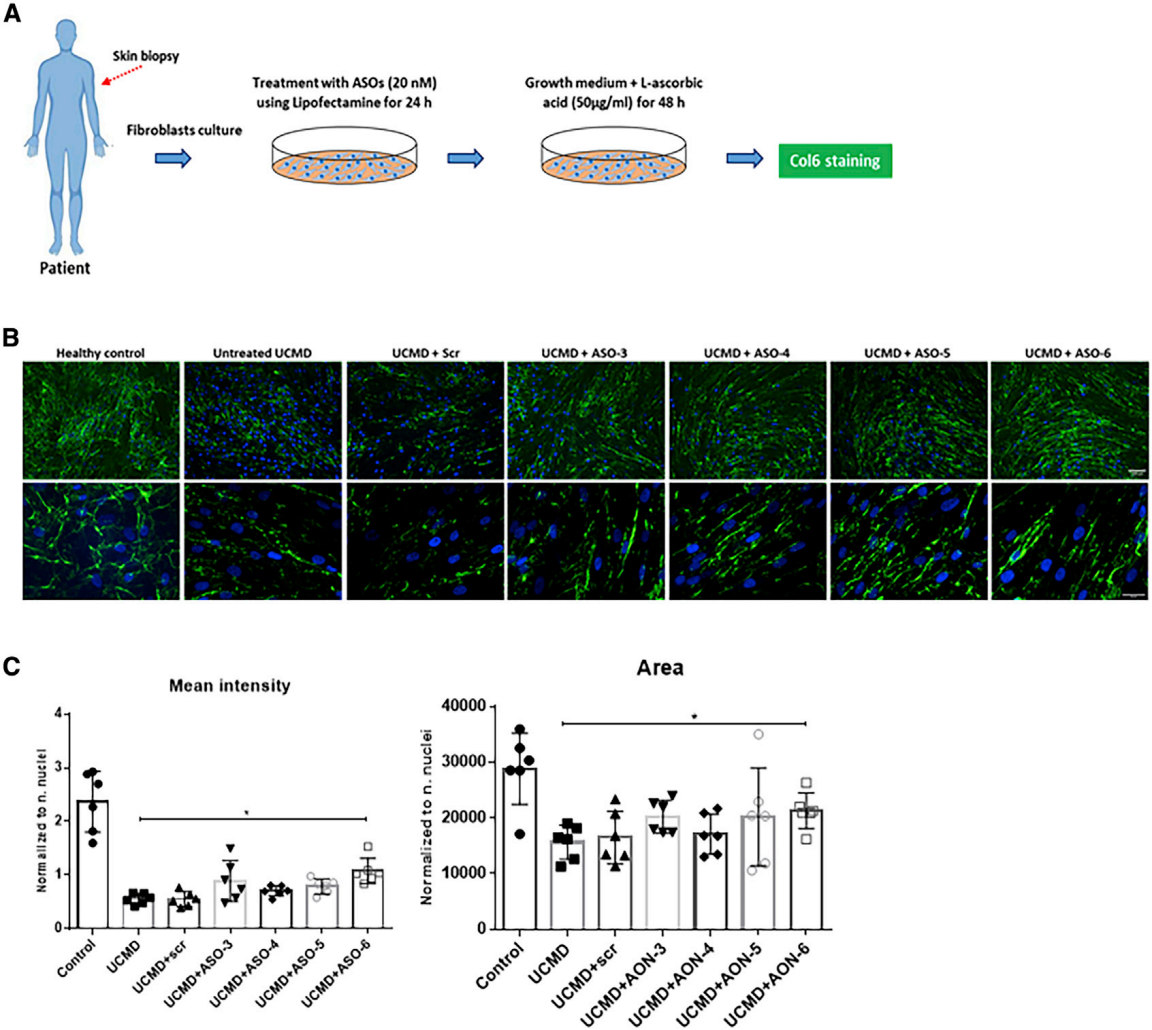
The Efficiency of ASOs on 2′-OMe Backbone in Restoring ECM Collagen VI Protein (A) Patient skin fibroblasts were treated with ASOs at 20 nM using Lipofectamine as transfection reagent. After 24 h, the transfection medium was replaced with growth medium containing l-ascorbic acid (50 μg/mL) for 48 h before being processed for collagen VI immunostaining. (B) Collagen VI protein in the ECM (in green) and nuclei (in blue) were displayed by immunofluorescence staining. Pictures were captured under fluorescence microscopy at 10× (upper panel) and 40× (lower panel) original magnification. Scale bars: 100 μm (upper panel) and 50 μm (lower panel), respectively. (C) Mean intensity and the area covered by collagen VI were quantified in fibroblasts treated with a single transfection of ASO-scr, ASO-3, ASO-4, ASO-5, and ASO-6 at 20 nM for 24 h followed by l-ascorbic acid (50 μg/mL) treatment for 48 h. Data represent mean ± SD from analysis of six individual field images acquired at 40× original magnification under fluorescence microscopy. Data were analyzed by one-way ANOVA and post-Bonferroni test (∗p ≤ 0.05; ∗∗p ≤ 0.01; ∗∗∗p ≤ 0.001).

In our recently reported study using PMOs, we identified an effective sequence region targeted by ASO-9, ASO-10, ASO-11, and ASO-12.^32^ Therefore, in this study, we focused on an additional region targeted by ASO-3, ASO-4, ASO-5, and ASO-6, evaluating their efficacy at protein level by immunofluorescence staining (Figure 3). Fibroblasts from UCMD patients were treated with the selected ASOs at 20 nM followed by 48-h incubation with l-ascorbic acid (50 μg/mL) (Figure 3A). As expected, untreated UCMD fibroblasts had reduced collagen VI protein expression in ECM compared with the healthy control. Moreover, instead of the linear and continuous collagen VI microfibrils in the healthy control, the untreated UCMD fibroblast cultures had collagen VI microfibrils that were discontinuous and speckled in appearance (Figure 3B). This abnormal appearance of the collagen VI matrix in untreated UCMD fibroblasts did not change after treatment with scrambled ASO (UCMD + scr ). With the exception of ASO-3, after a single treatment with ASO-4, ASO-5, and ASO-6 at 20 nM, the collagen VI acquired a much more continuous and smooth appearance, similar to the normal control (Figure 3B). The intensity of collagen VI immunoreactivity in the ECM also appeared to be increased by visual inspection, compared with the untreated or scrambled treatment. Semi-quantification of collagen VI mean intensity confirmed a significant increase of collagen VI deposition in the ECM after ASO-6 treatment (p = 0.0201), compared with the untreated UCMD, although still significantly less than the normal control level. Next, we measured the area covered by positive collagen VI immunostaining to semi-quantify the protein distribution. As expected, the increase in the mean intensity in ASO-6-treated UCMD fibroblasts was accompanied by a significant increase in the area covered by the protein, compared with the untreated UCMD fibroblasts (p = 0.0416) (Figure 3C).

Based on their effects on ECM collagen VI restoration and microfibril structure, and their striking efficiency in inducing pseudo-exon skipping at the RNA level, we selected ASO-5 and ASO-6 for further validation. In addition, a shorter 20-mer antisense sequence, ASO-5/6, which overlapped between ASO-5 and ASO-6, was included for further evaluation and compared with the efficiency of the previously published ASO-9 in 2′-MOE chemistry.

### 2′-MOE ASOs Correct the Mutant Transcripts in a Dose-Dependent Manner

**Figure 4:**
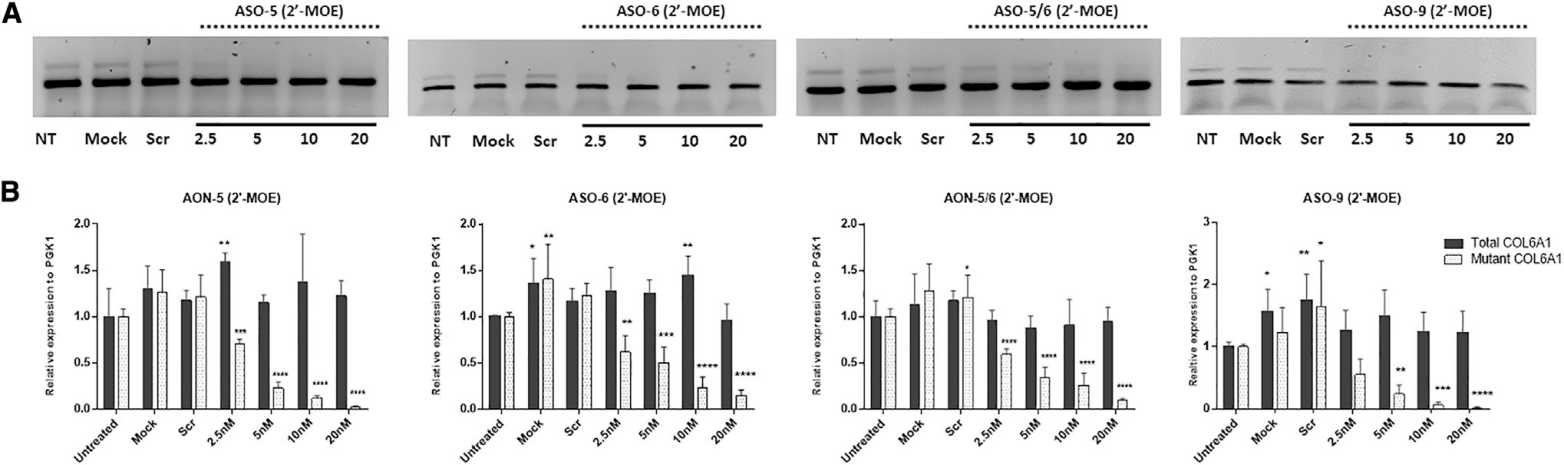
The Efficiency of ASOs in 2′-MOE Backbone in Pseudo-Exon Skipping at RNA Level (A) Representative picture of PCR products from fibroblasts treated with ASO-5, ASO-6, ASO-5/6, or ASO-9 in 2′-MOE backbone for 24 h with Lipofectamine transfection. (B) Quantitative real-time PCRs of total *COL6A1* and mutant *COL6A1* transcripts were performed in RNA samples collected from four patient skin fibroblasts treated with ASO-5, ASO-6, ASO-5/6, or ASO-9 at concentrations ranging from 2.5 to 20 nM. Data are presented as mean ± SD. Data were analyzed by one-way ANOVA and post-Bonferroni test (∗p ≤ 0.05; ∗∗p ≤ 0.01; ∗∗∗∗p ≤ 0.0001).

ASO-5 (25-mer), ASO-6 (25-mer), ASO-9 (25-mer), and ASO-5/6 (20-mer) were synthesized in 2′-MOE chemistry (Table 1). Dose-response studies were performed at concentrations ranging from 2.5 to 20 nM. The four UCMD patient skin fibroblasts were treated using Lipofectamine as transfection reagent for 24 h, followed by RNA extraction, PCR, and quantitative real-time PCR (Figure 4). There was a suppression of pseudo-exon inclusion in the mature transcripts in a dose-dependent manner (Figure 4A). Pseudo-exon skipping ranged from 29.1% (±4.8) after 2.5 nM to 97.6% (±1.1) after 20 nM ASO-5 treatment, from 38.1% (±17.8) after 2.5 nM to 85.3% (±6.3) after 20 nM ASO-6 treatment, from 44.7% (±24) after 2.5 nM to 98% (±1) after 20 nM ASO-9 treatment, and 42.6% (±6.1) after 2.5 nM to 90.2% (±2.7) after 20 nM ASO-5/6 treatment (Figure 4B). For all four ASOs, 20 nM elicited the greatest skipping effect on the pseudo-exon without affecting the expression of the wild-type transcripts as determined by quantitative real-time PCR (Figure 4B). This concentration was therefore used for subsequent studies at the protein level.

### 2′-MOE ASOs Restore Collagen VI Protein and Structure in ECM

**Figure 5:**
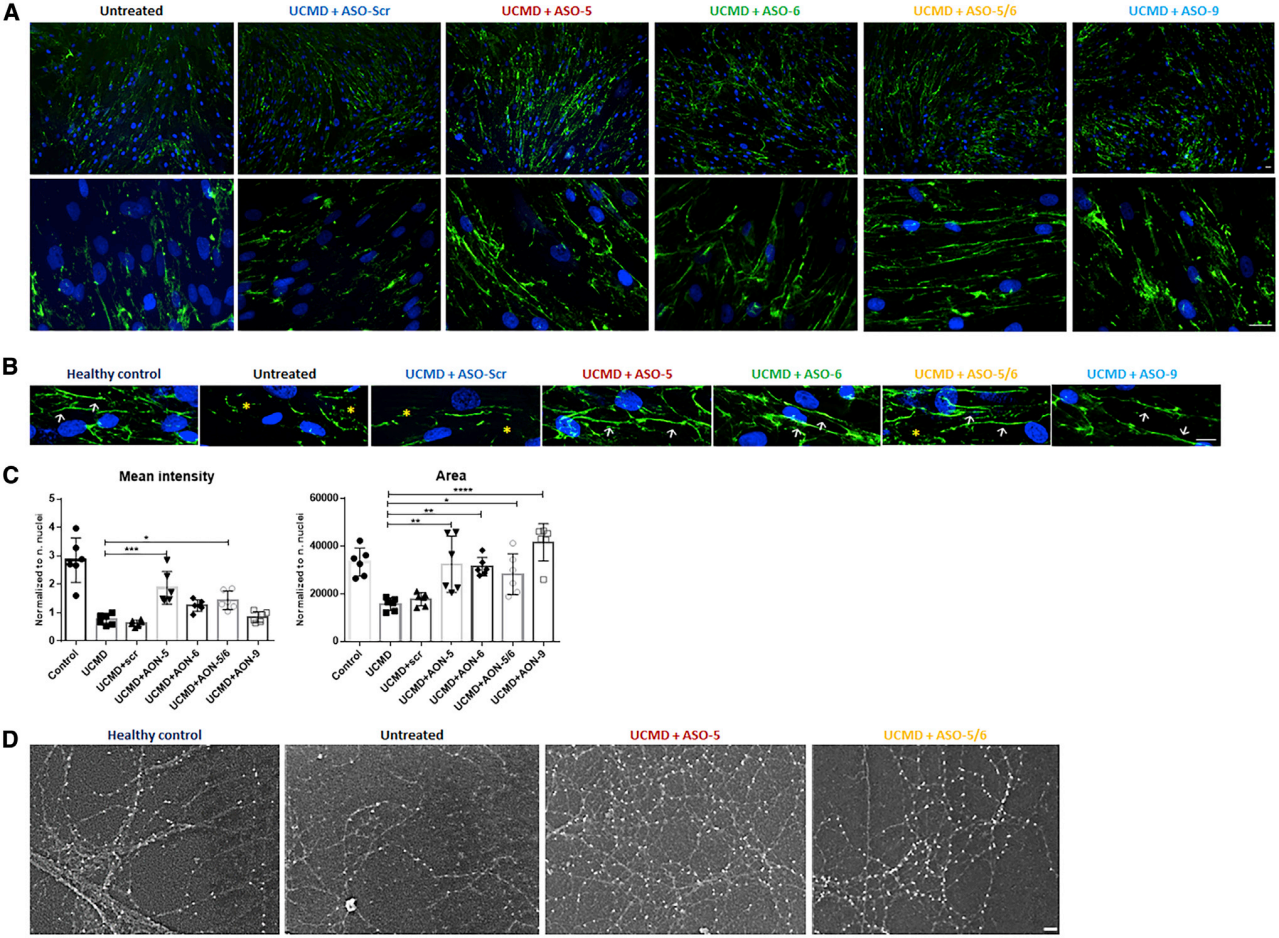
The Efficiency of ASOs on 2′-MOE Backbone in Restoring ECM Collagen VI Protein (A) Representative images of immunofluorescence staining of collagen VI protein (in green) and nuclei (in blue) in patient skin fibroblasts treated with 2′-MOE ASOs. Pictures were captured under fluorescence microscopy at 10× (top panel) and 40× (lower panel) original magnification. Scale bars: 100 μm for 10× magnification and 50 μm for 40× original magnification. (B) The structure of ECM collagen VI expression in fibroblasts from healthy control and UCMD patients treated with 20 nM ASOs. Images were captured under confocal microscope. In healthy condition, collagen VI forms the liner microfibrils (white arrow) in ECM, while in UCMD patients the linear structure is replaced with discontinuous and speckled microfibrils (yellow asterisk). Treatment of ASO-5, ASO-6, ASO-5/6, or ASO-9 restored the collagen VI deposition pattern to linear microfibrils. Scale bar: 10 μm. (C) Mean intensity and the area covered by collagen VI were quantified in fibroblasts treated with a single transfection of ASO-scr, ASO-5, ASO-6, ASO-5/6, and ASO-9 at 20 nM. Data represent mean ± SD. Data were analyzed by one-way ANOVA followed by post-Bonferroni test (∗p ≤ 0.05; ∗∗p ≤ 0.01; ∗∗∗p ≤ 0.001). (D) The beaded microfibril network of collagen VI protein was visualized by scanning electron microscopy in UCMD fibroblasts treated with 20 nM ASO-5 or ASO-5/6. Scale bar: 100 nm.

Collagen VI matrix staining was studied in four fibroblast cell lines carrying a c.930+189C > T mutation after treatment with the four 2′-MOE ASOs at 20 nM. In all cell lines, there was an increase in collagen VI deposition in ECM as determined by immunofluorescence analysis after a single treatment with all four ASOs. Compared with the untreated (UCMD) or scrambled ASO treated (UCMD + scr) fibroblasts, the ASO treatment gave not only an increase in collagen VI secretion in ECM but also an improvement in microfibril structure (Figures 5A and 5B).

In order to evaluate the structure of ECM collagen VI in response to ASO treatment, we visualized collagen VI immunoreactivity in fibroblast cultures by confocal microscopy. In the untreated or scrambled-treated UCMD fibroblasts, the structure of collagen VI microfibrils in the ECM was discontinuous, compared with the linear microfibrils in the healthy control. After a single treatment of ASO-5, ASO-6, ASO-9, or ASO-5/6, the discontinuous pattern reverted to a more linear fashion, similar to the healthy control, suggesting that the suppression of the pseudo-exon at the transcript level rapidly translates to exclusion of the mutant peptide from collagen VI tetramers, allowing for normalization of the matrix (Figure 5B). The area covered by collagen VI immunoreactivity was significantly increased after a single treatment of any of the four ASOs tested, but the mean fluorescence intensity was significantly increased only for ASO-5 and ASO-5/6 (Figure 5C).

The microfibril structure of collagen VI in the ECM was further characterized by scanning electron microscopy (SEM). The conformation of collagen VI-positive microfibrils was investigated after a single treatment of the shorter ASO-5/6 and one ASO of 25-mer long (ASO-5) at 20 nM. The untreated cultures had a notable reduction in collagen VI-positive microfibrils, with a disorganized network compared with the healthy control. SEM analysis of the untreated UCMD fibroblasts clearly showed the inability of the mutant tetramers to assemble, leading to a reduction of microfibril formation and disrupting the organization of the typical collagen VI microfibril network. Interestingly, after ASO treatment, the pattern of the collagen VI network was markedly improved. A single treatment of ASO-5 or ASO-5/6 led to an increase in ECM deposition, together with a significant restoration of regularly beaded microfibrils network (Figure 5D).

### 2′-MOE ASOs Were Efficiently Taken up in Patients’ Fibroblasts in the Absence of Transfection Reagent

**Figure 6:**
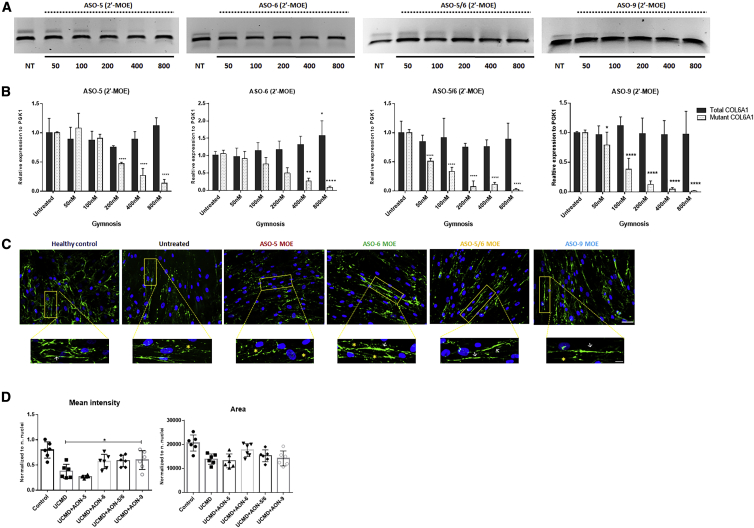
The Efficiency of ASOs in Pseudo-Exon Skipping by Gymnotic Delivery (A) Representative images of PCR products from fibroblasts treated with ASO-5, ASO-6, ASO-5/6, or ASO-9 in 2′-MOE backbone for 4 days without the use of any transfection reagent. (B) Quantitative real-time PCRs of total *COL6A1* and mutant *COL6A1* transcripts were performed in RNA samples collected from four patient skin fibroblasts treated with ASO-5, ASO-6, or ASO-5/6 at concentrations ranging from 50 to 800 nM. Data are presented as mean ± SD. Data were analyzed by one-way ANOVA and post-Bonferroni test (∗p ≤ 0.05; ∗∗p ≤ 0.01; ∗∗∗p ≤ 0.001). (C) Representative images of ECM collagen VI protein expression in patient skin fibroblasts treated with ASO-5, ASO-6, ASO-5/6, and ASO-9 at 800 nM for 4 days with gymnotic delivery. Collagen VI protein (in green) and nuclei (in blue) are displayed by immunofluorescence staining. The linear collagen VI microfibrils are indicated by white arrows and the discontinuous collagen VI microfibrils by yellow asterisks. Scale bar: 50 μm. (D) Mean intensity and the area covered by collagen VI were quantified in fibroblasts after the gymnotic delivery of ASO-5, ASO-6, ASO-5/6, and ASO-9 at 800 nM. Data represent mean ± SD. Data were analyzed by one-way ANOVA and post-Bonferroni test (∗p ≤ 0.05; ∗∗p ≤ 0.01; ∗∗∗p ≤ 0.001).

The delivery of ASO-5, ASO-6, ASO-9, and ASO-5/6 in 2′-MOE in cultured skin fibroblasts was also tested in the absence of any transfection reagents (gymnotic delivery) to better reflect an *in vivo* situation where transfection agents cannot be used. A range of concentrations between 50 and 800 nM was examined. 2′-MOE ASOs were added to fibroblast growth medium and left for 4 days without changing the medium. On the fifth day, RNA extraction, PCR, and quantitative real-time PCR were performed (Figures 6A and 6B). Pseudo-exon skipping occurred in ASO-treated fibroblasts in a dose-dependent manner. For ASO-5, pseudo-exon skipping occurred at 200 nM (53.1% ± 2.3%) and reached 86.4% (±5.6%) at 800 nM. For ASO-6, pseudo-exon skipping was 50.3% (±15.3%) at 200 nM and 91.5% (±3.2) at 800 nM. For ASO-9, pseudo-exon skipping occurred at 100 nM (62% ± 19%) and reached 98% (±0.7) at 800 nM. Moreover, the skipping efficiency was markedly higher in cells treated with ASO-5/6, with exon skipping detected at concentrations as low as 50 nM (48.8% ± 4.5%) and reaching 97.5% (±1.4%) at 800 nM (Figure 6B).

The efficiency of 2′-MOE ASOs on restoring ECM collagen VI via gymnotic delivery was further evaluated at the protein level. UCMD fibroblasts were treated with ASO-5, ASO-6, ASO-5/6, or ASO-9 in 2′-MOE at 800 nM for 4 days, followed by l-ascorbic acid treatment for 48 h. ECM collagen VI protein was evaluated by immunostaining in fibroblast cultures. There was no significant improvement in collagen VI deposition or microfibril structure in ASO-5-treated UCMD fibroblasts, when compared with the untreated cells, while an obvious increase in ECM collagen VI deposition and improved linear microfibrils formation occurred in fibroblasts treated with ASO-6, ASO-9, or ASO-5/6 (Figures 6C and 6D).

## Discussion

UCMD is the severe form of COL6-CMD caused by recessively or dominant-negatively acting mutations in any of the three genes encoding collagen VI. Dominant-negative mutations occur in more than half of UCMD cases.

There are two potential therapeutic approaches to reduce the dominant-negative effect of the mutant collagen VI α chain by harnessing antisense technology. We have previously reported an approach using gapmer ASO to specifically silence the mutant RNA transcript by activating RNase H, based on the fact that haploinsufficiency is not associated with any disease phenotype.^27^^,^^30^^,^^31^

Another approach is to use ASO to modulate splicing by interfering with pre-mRNA splicing events.^32^ This splice switching approach has been successfully applied to other neuromuscular diseases for both exon inclusion and exclusion, including nusinersen for spinal muscular atrophy in which ASO induced *SMN2* exon 7 inclusion, and eteplirsen for Duchenne muscular dystrophy, where skipping of exons neighboring out-of-frame deletions is capable of restoring the reading frame.^34^, ^35^, ^36^ In this study, we investigated an exon-skipping strategy to exclude the pseudo-exon splicing resulting from the *de novo* c.930+198C > T heterozygous mutation in the *COL6A1* gene (Figure 1A).^32^^,^^33^ Arguably, pseudo-exon exclusion is the perfect target for exon-skipping approaches using ASO, because the resulting transcripts will revert to wild type.

We recently reported the identification of PMOs targeting this mutation that led to significant pseudo-exon skipping and subsequently restored collagen VI matrix assembly in ECM.^32^ In order to find the optimal candidate ASO capable of efficiently skipping the pseudo-exon from the mutated *COL6A1* transcripts, we here conducted a more systematic screening of ASOs designed to cover the entire pseudo-exon and its flanking 5′ and 3′ splice sites using different backbone chemistry (Figures 1B and 1C). In total, 15 ASOs were tested in fibroblast cell lines established from four patients carrying the *de novo* c.930+198C > T mutation. There were two regions in the pseudo-exon, where ASOs elicited efficient exon skipping (Figure 2C). The sequence region covered by ASO-9, ASO-10, ASO-11, and ASO-12 is consistent with our previous publication on PMOs.^32^ In this study, we focused on a different region targeted by ASO-3, ASO-4, ASO-5, and ASO-6, where most of the predicted splicing enhancer motifs are located (Figure 1B). The efficiency of these ASOs was initially evaluated in 2′-OMe chemistry. Based on the evaluation of all 2′-OMe ASOs at RNA and protein levels (Figures 2C and 3B), ASO-5 and ASO-6 were selected for further validation in 2′-MOE, a well-investigated antisense chemistry currently used in nusinersen for the treatment of spinal muscular atrophy. Together with these two ASOs, a shorter 20-mer ASO-5/6 with sequence shared between ASO-5 and ASO-6 and the already published ASO-9 were additionally designed in 2′-MOE for further experiments.

By using Lipofectamine as a transfection agent, we achieved a significant decrease in the pseudo-exon transcripts in a dose-dependent manner, reaching over 90% efficiency at 20 nM after ASO-5, ASO-6, or ASO-9 treatment (Figure 4). This was further confirmed at the protein level. A single treatment of ASOs at 20 nM led to significantly increased deposition of collagen VI in the ECM and a rescue in microfibril structure (Figure 5A). The effect of ASO treatment was also seen in the ultrastructural network of beaded microfilaments that collagen VI forms in ECM by scanning electron microscopy. The disrupted networks observed in the patient fibroblasts were rectified by a single 2′-MOE ASO treatment (Figure 5D).

Transfection reagent-free uptake or gymnosis has recently been recommended as a method for *in vitro* ASO evaluation.^37^ In the absence of any transfection reagent and the presence of normal culture medium, this situation may mimic the *in vivo* delivery of ASO more closely than the forced delivery by Lipofectamine in low-serum transfection medium.^37^, ^38^, ^39^ Using this technique, all four ASOs in 2′-MOE chemistry gave a significant restoration of collagen VI at RNA level at nanomolar concentrations. The shorter 20-mer ASO-5/6 and ASO-9 were the most efficient, followed by the 25-mer ASO-6 and ASO-5 (Figure 6B). The effective concentration (EC_50_) of the 20-mer ASO-5/6 for gymnotic delivery was 50 nM, compared with 100 nM for the 25-mer ASO-9 and 200 nM for ASO-5 and ASO-6. This result suggests that a shorter 2′-MOE ASO (20-mer) might be more efficient than longer ASOs (25-mer) for gymnosis delivery. The moderate splice-modulating efficacy of ASO-5 that we observed at both the RNA and the protein levels may also be due to its guanine-cytosine (GC) content that is higher than in the other ASOs tested (76% versus 55% on average). Although GC content can strengthen the binding affinity, a too-high content (i.e., 76% for ASO-5) is, however, far beyond the acceptable range and may affect its chemical stability and hence efficacy in cells.^40^ Importantly, in cells treated with a wide range of concentrations (50–800 nM), although the mutant transcripts were dramatically reduced in a dose-dependent manner, there were no changes in the expression of total collagen VI transcripts. Our results indicate that these splice-switching ASOs are able to specifically correct the aberrant splice event and convert the mutant transcripts to wild-type products by keeping the total *COL6A1* mRNA levels unchanged. This also provides the indirect evidence that no gene silencing effect was induced by these ASOs.

The success of antisense drug development depends not only on the identification of the optimal sequences but also on the efficient engagement with the target cell/organ *in vivo*. Increasing numbers of chemical modifications and novel nucleotide technologies have been developed to improve the *in vivo* efficacy of RNA therapy with reduced toxicity and enhanced biodistribution.^41^ In COL6-related UCMD, the target cell population is the interstitial fibroblasts in skeletal muscle.^13^ PMO and 2′-MOE chemistries are efficient in modifying pre-mRNA splicing and are used in US Food and Drug Administration (FDA)-approved ASO drugs in other neuromuscular disorders.^34^, ^35^, ^36^ However, the biodistribution of these ASOs in disease-relevant muscle interstitial fibroblasts after systemic delivery is unknown. It is also not clear whether the restoration of collagen VI in skeletal muscle will reverse muscle function or only slow down disease progression, and what minimum levels of mutant collagen VI need to be corrected in order to show a clinically meaningful therapeutic benefit. Nevertheless, observation in patients with somatic mosaicism for dominant-negative mutations, who are less symptomatic compared with full expression of the mutations, indicates that incomplete correction of the mutation may also be beneficial.^42^

In conclusion, in this study, we performed a systematic screening of exon-skipping ASOs in correcting the aberrant splicing event caused by the recurrent dominant mutation c.930+198C > T in intron 11 of *COL6A1* gene. We identified two additional lead ASOs in 2′-MOE chemistry, which, following gymnotic delivery at nanomolar concentrations, efficiently restored functional collagen VI protein expression in the ECM of patient fibroblasts *in vitro*. Our findings provide further evidence to support ASO exon skipping as a therapeutic approach for a group of COL6-CMD patients carrying the c.930+198C > T mutation. Further studies on *in vivo* efficacy and safety in a suitable mouse model are needed before this approach can be eventually translated to clinical trials.

## Materials and Methods

### Patients

All patients and controls in this study provided written informed consent for skin biopsy samples, following the Declaration of Helsinki protocols. Fibroblast cultures from four patients and two controls (no pathogenic variants in all *COL6A* genes) were established from skin biopsies.

### Cell Culture

Fibroblasts were supplied by the MRC Centre for Neuromuscular Disease Biobank London (REC reference number 06/Q0406/33). Fibroblasts were grown in Dulbecco’s modified Eagle’s medium (DMEM; Invitrogen) supplemented with 10% fetal bovine serum (FBS; Life Technologies) and 1% penicillin-streptomycin (Sigma) at 37°C and 5% CO_2_. At approximately 80% confluence, cells were split and all experiments were performed on cells at passage 4–11.

### *COL6A1* Mutation and ASOs Design

The mutation studied in this project is a recurrent *de novo COL6A1* deep intronic mutation (c.930+189C > T) in heterozygous state.^32^ This variant causes the insertion of an in-frame pseudo-exon, which consists of 72 bp of intron 11. ASOs were designed to target the entire pseudo-exon and its flanking 5′ and 3′ splice sites, using “RNA-tiling approach” (Figures 1B and 1C). ASOs were synthesized in 2′-OMe or 2′-MOE chemistries (Eurogentec) (Table 1).

### ASO Treatment

ASO treatment was performed with or without transfection reagents (gymnotic delivery). Lipofectamine 2000 (Life Technologies) was used as a transfection reagent with Opti-MEM (Life Technologies). For transfection, cells were seeded in a six-well plate at a density of 2 × 10^5^ per well in growth medium in order to reach 80% confluence on the following day. Cells were then transfected for 24 h in Opti-MEM according to the manufacturer’s instructions.

For gymnotic delivery, cells were seeded in a six-well plate at a density of 1 × 10^5^ per well in growth medium. The following day, ASOs were added according to the desired final concentration in the growth medium and incubated for 4 days in growth medium before carrying out further experiments. All ASO treatment experiments performed above were conducted in fibroblast lines from four UCMD patients.

### RNA Extraction, One-Step PCR, and Gel Electrophoresis

For the evaluation at RNA level, after 24 h of transfection or after 4 days of gymnotic delivery, cells were harvested for total RNA extraction. Total RNA was extracted from fibroblasts using RNeasy mini kit (QIAGEN) according to the manufacturer’s instructions. Quality and quantity of RNA was assessed by NanoDrop spectrophotometer (Thermo Fisher Scientific). To amplify *COL6A1* transcripts in pre- and post-treated samples, we used 200 ng total RNA in One-Step RT-PCR (QIAGEN). A pair of primers were used to amplify both wild-type (109 bp) and mutant (181 bp) transcripts (forward: 5′-TAC CAG GGA ATG AAG GGA GA-3′ and reverse: 5′-GTC CTT GAA TGC CGT CAA AC-3′). The products were amplified using the following protocol: the initial reverse-transcription reaction at 50°C for 30 min and the PCR activation step at 95°C for 15 min followed by denaturation at 94°C for 30 s, annealing at 60°C for 30 s, and extension at 72°C for 60 s. The last three steps were run for a total of 30 cycles finishing at 72°C for 10 min as the final extension. The amplicons were then run on a 1.5% agarose gel and visualized under the Gel Doc XR imaging system (Bio-Rad).

### Reverse Transcription and Quantitative Real-Time PCR

Superscript III reverse transcriptase kit (Life Technologies) was used for first-strand cDNA synthesis using 1 μg total RNA according to the manufacturer’s instructions. Quantitative real-time PCR analysis was performed using the Takyon ROX Probe qPCR kit (Eurogentec) with 20 ng cDNA as template. Primers and probes for the detection of total *COL6A1* transcripts (wild type + mutant) and mutant *COL6A1* transcripts were specifically designed as described previously.^32^ StepOne real-time PCR system (Applied Biosystems) was used for quantitative real-time PCR and analysis using the following run method: holding stage at 50°C for 2 min and 95°C for 10 min followed by 40 cycles of denaturation at 95°C for 15 s and annealing extension at 60°C for 1 min. Four biological replicates (from four UCMD patients) and two technical replicates were used for data analysis. The relative quantification was measured by ΔΔCt method using *phosphoglycerate kinase 1* gene (*PGK1*) as the endogenous reference gene.

### Collagen VI Immunofluorescence

Cells were seeded on collagen-precoated glass coverslips (Corning) in a six-well plate. After either 24 h of transfection or 4 days of gymnotic delivery, the medium was replaced with growth medium containing l-ascorbic acid (50 μg/mL) for 48 h to promote the secretion of collagen VI protein in ECM.^43^ Cells were fixed with 4% paraformaldehyde for 10 min at room temperature (RT) followed by 1-h incubation in blocking buffer with 5% goat serum in PBS (Sigma-Aldrich). Collagen VI was probed by mouse anti-human antibody (MAB1944, 1:2,000 dilution; Merck Millipore) diluted in blocking solution for 1 h at RT. Coverslips were washed three times with PBS and incubated with goat anti-mouse antibody conjugated to Alexa Fluor 488 diluted in blocking solution (A11029, 1:500 dilution; Invitrogen) at RT for 1 h. After three washes in PBS, nuclei were stained with Hoechst 33342 (1:2,000 dilution; Invitrogen) diluted in PBS for 3 min and rinsed with PBS. Coverslips were then mounted with hydromount (National Diagnostics). Cells were visualized with either a LSM710 Zeiss Confocal microscope or Leica DMR fluorescence microscope. Images were captured using Zen black or MetaMorph software, with a fixed exposure setting for all the slides.

The semi-quantification of collagen VI fluorescence signals was analyzed using ImageJ version 1.52p (https://imagej.nih.gov/ij/). Two parameters were measured: collagen VI mean intensity and the area covered by collagen VI. For each sample, six fields of view acquired with the same setting were analyzed. A rolling ball radius of 50 pixels and the same threshold were set for all images. The number of nuclei was used to normalize the two parameters.

### Scanning Electron Microscopy

Cells were seeded on glass coverslips (Corning) at 2 × 10^5^ density per well in a six-well plate. After 24 h of transfection, transfection medium was replaced with growth medium containing l-ascorbic acid (50 μg/mL) for 48 h. Cells were fixed with 4% paraformaldehyde for 10 min at RT followed by 1-h incubation with 5% goat serum blocking solution (Sigma-Aldrich) and 0.1% Triton X-100 (Sigma-Aldrich) for cell permeabilization. Blocking solution in PBS was filtered with a 22-μm filter (Millex GP) before use. After incubation with blocking solution, collagen VI was probed by mouse anti-human antibody (MAB1944, 1:2000 dilution; Merck Millipore) diluted in blocking solution for 1 h at RT. Coverslips were washed three times with PBS containing 0.1% Triton X-100 and incubated with secondary anti-mouse IgA conjugated with 10 nm gold (EM.GAMA10, 1:200 dilution, BBI solution) at RT for 90 min. Incubation with secondary antibody was followed by three washes in PBS containing 0.1% Triton X-100 and fixation with 1% glutaraldehyde (Sigma-Aldrich) in PBS at RT for 10 min. The coverslips were postfixed in 1% OsO_4_/1.5% potassium ferrocyanide in 0.1 M cacodylate buffer at 3°C for 40 min. After rinsing with dH_2_O, specimens were dehydrated in a graded ethanol-water series to 100% ethanol. The samples were critical point dried using CO_2_. The coverslips were mounted on aluminum stubs using silver dag. The mounted samples were coated with a thin layer of carbon (approximately 7 nm thick) using a Gatan ion beam coater. The coverslips were imaged by combining the secondary and back-scattered signal using Jeol 7401 FEG SEM.

### Statistics

GraphPad Prism version 6.0 was used for graphs and statistical analysis. Data were analyzed using one-way ANOVA and post Bonferroni test to determine statistical significance. Data were presented as mean ± standard deviation (mean ± SD). Differences were considered to be statistically significance at ∗p ≤ 0.05; ∗∗p ≤ 0.01; ∗∗∗∗p ≤ 0.0001.

## Author Contributions

H.Z., F.M., and S.A. conceptualized and designed the study and experiments; H.Z. designed the antisense oligomers; S.A. performed and analyzed the experiments; P.A. established fibroblast lines from patients’ skin biopsies; M.T performed electron microscopy (EM) studies; S.A., H.Z. V.B., C.G.B., and F.M. wrote the manuscript.

## Conflicts of Interest

C.G.B., V.B., and F.M. share a patent related to the diagnosis of the mutation described in this study and to a method for treating it (PCT/US2017/040726-WO 2018/009547A1).
